# Analysis of chromosomal aberrations and recombination by allelic bias in RNA-Seq

**DOI:** 10.1038/ncomms12144

**Published:** 2016-07-07

**Authors:** Uri Weissbein, Maya Schachter, Dieter Egli, Nissim Benvenisty

**Affiliations:** 1 The Azrieli Center for Stem Cells and Genetic Research, Department of Genetics, Silberman Institute of Life Sciences, The Hebrew University, Jerusalem 91904, Israel; 2 The New York Stem Cell Foundation Research Institute, New York, New York 10032, USA; 3 Naomi Berrie Diabetes Center, Columbia University, New York, New York 10032, USA; 4 Department of Pediatrics, College of Physicians and Surgeons, Columbia University, New York, New York 10032, USA

## Abstract

Genomic instability has profound effects on cellular phenotypes. Studies have shown that pluripotent cells with abnormal karyotypes may grow faster, differentiate less and become more resistance to apoptosis. Previously, we showed that microarray gene expression profiles can be utilized for the analysis of chromosomal aberrations by comparing gene expression levels between normal and aneuploid samples. Here we adopted this method for RNA-Seq data and present eSNP-Karyotyping for the detection of chromosomal aberrations, based on measuring the ratio of expression between the two alleles. We demonstrate its ability to detect chromosomal gains and losses in pluripotent cells and their derivatives, as well as meiotic recombination patterns. This method is advantageous since it does not require matched diploid samples for comparison, is less sensitive to global expression changes caused by the aberration and utilizes already available gene expression profiles to determine chromosomal aberrations.

## Abstract (web summary)

Chromosomal aberrations can be detected by global gene expression analysis. Here, the authors report eSNP-Karyotyping, a new method that can detect chromosomal aberrations by measuring the ratio of expression between the two alleles without comparison to a matched diploid sample.

## Graphical abstract

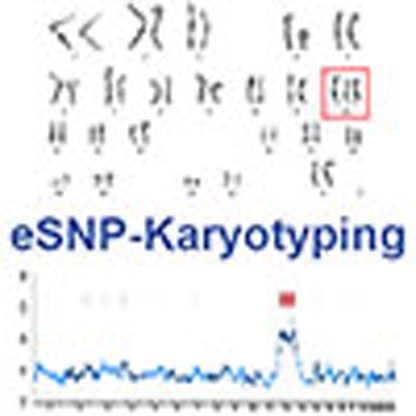

Human pluripotent stem cells (hPSC) acquire chromosomal abnormalities during their derivation and their propagation in culture^1^. These aberrations might affect cellular behaviours such as the cell cycle, apoptosis resistance, tumorigenicity and differentiation capabilities due to changes in expression levels of various genes^1^,^2^,^3^,^4^,^5^. Hence, cells carrying certain aberrations take over the culture due to positive selective pressures^2^,^5^,^6^. Notably, this selective process, which is not unique to hPSC as it also occurs in other cell types in humans and other mammals^7^,^8^,^9^, may affect genetic screens, basic research studies and future regenerative medicine^1^.

Chromosomal aberrations are traditionally detected using methods that require accessibility to the genetic material of the cells. These methods include cytogenetic analysis of metaphase chromosome spreads using Giemsa banding or spectral karyotyping (SKY), or analysis of the DNA content of the cells using techniques such as array-comparative genomic hybridization (aCGH), single-nucleotide polymorphism (SNP) arrays and whole-genome sequencing (WGS)^10^. Each of these methods can successfully detect chromosomal aberrations. Previously, we presented a methodology, named e-Karyotyping, for studying genomic instability by analysis of global gene expression using microarray data sets^6^,^7^,^10^. This method is based on comparison of gene expression levels along chromosomes by comparing the sample of interest and a matched diploid sample, to look for regional differences in gene expression. e-Karyotyping analysis does not require accessibility to chromosomal or DNA material, and can be performed on any gene expression microarray analysis. A prerequisite of e-Karyotyping is the availability of the gene expression profile of normal diploid samples of the exact cell type for comparison^10^.

Here we initially adopted this methodology for global gene expression analysis obtained from RNA-Seq data, and then developed a new strategy to analyse genomic integrity based on the expression of transcripts with allele bias. This method enables a reliable and fast analysis of genomic integrity, without the need for comparison to a matched diploid sample.

## Results

### Applying e-Karyotyping to RNA-Seq data

**Figure 1:**
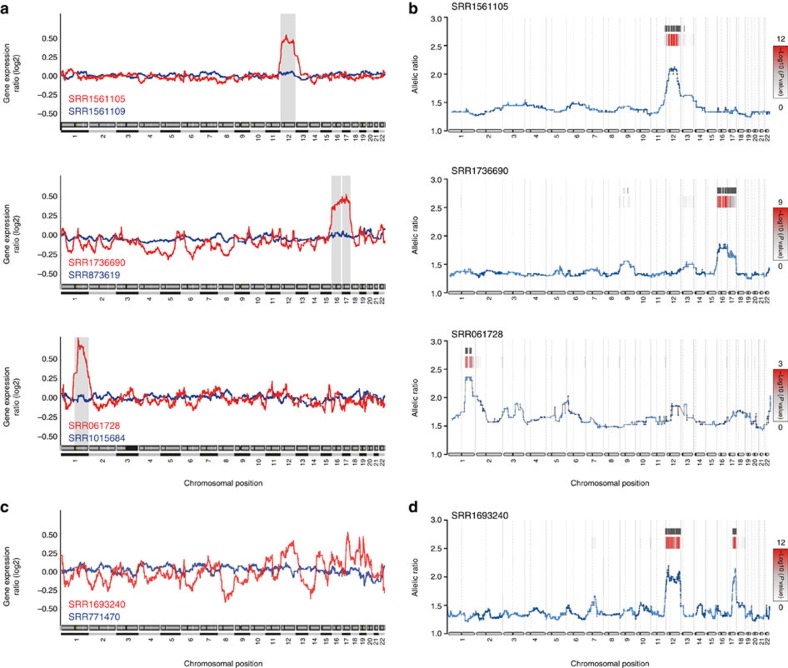
Detection of chromosomal duplications using RNA-Seq data. (**a**) e-Karyotyping analysis of samples from RNA-Seq studies. Shown are moving average plots of representative examples of chromosomal aneuploidies in pluripotent and pluripotent-derived cells. The grey background represents statistically significant aneuploidy as recognized by the piecewise constant fit algorithm. (**b**) eSNP-Karyotyping of the aberrant samples shown in **a**. Colour bars represent the FDR-corrected *P* value. Positions with a *P* value lower than 0.01 are marked by a black line. (**c**) Two representative samples from the e-Karyotyping analysis for PSC-derived pancreatic progenitor cells. (**d**) eSNP-Karyotyping for the red sample analysed in **c**.

To adapt e-Karyotyping for RNA-Seq data, we collected multiple RNA-Seq data sets of human pluripotent or pluripotent-derived cells from the Sequence Read Archive (SRA) database (http://www.ncbi.nlm.nih.gov/Traces/sra/)^11^ (Supplementary Table 1), aligned the reads to the genome using TopHat2 (ref. 12), and retrieved the normalized fragments per kilobase of transcript per million mapped reads (FPKM) values for each gene using Cufflinks^13^. Next, we generated a table of the merged expression values and divided each gene expression level by the median expression levels across all samples, as previously described for microarray intensity values^6^,^10^. To reduce noise, we discarded transcripts that were unexpressed (less than a FPKM value of 1) in more than 20% of the samples, from further analysis. In addition, we discarded the 10% most variable transcripts (see Methods). Using a piecewise constant fit algorithm^14^ with a set of defined parameters (see Methods) we could detect regional biases in gene expression. We identified samples with trisomy 12, and 16 together with 17, as well as a sample with trisomy 1q (Fig. 1a and Supplementary Fig. 1), which are easily visualized using moving average plots. These aberrations are well-known recurrent changes in pluripotent cell cultures due to positive selection (except trisomy 16)^6^.

### Detection of chromosomal aberrations using eSNP-Karyotyping

**Figure 2:**
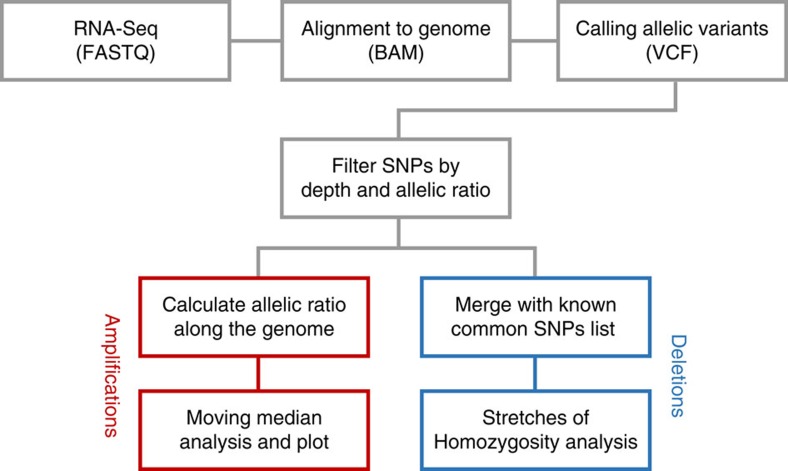
eSNP-Karyotyping data analysis workflow. Schematic overview of the analysis to detect chromosomal aberrations by determining allelic ratio in the RNA-Seq data.

In addition to gene expression levels, RNA-Seq can provide information about the underlying DNA sequence. Most genes are expressed from both alleles at the same levels (except for cases of monoallelic expression such as parental imprinting)^15^, especially when RNA is extracted from a population of cells^16^. We reasoned that in cases of chromosomal duplications, a deviation from the expected 1:1 ratio between the alleles, localized to the duplicated region, should be detected. Therefore, we developed a workflow that first calls SNPs from the RNA-Seq data using the GATK HaplotypeCaller programme^17^. This tool calls for probable variants from next-generation sequencing data, and returns the reads number for each variant. Next, we filtered out SNPs below a threshold coverage of 20 reads, and SNPs with a frequency below 0.2 of the less-expressed allele, to eliminate biases of the library preparation, sequencing errors or low reading depth. We then ordered the remaining SNPs according to their chromosomal location and calculated for each SNP the number of reads ratio between the more-expressed allele (major allele) and the less-expressed one (minor allele). We term this method expressed-SNP-karyotyping (eSNP-Karyotyping) (Fig. 2). An R package of the new methodology is available for download from GitHub (https://github.com/BenvenLab/eSNPKaryotyping). To evaluate our method, we first tested it on the samples analysed by e-Karyotyping. While the diploid samples produced a constant allelic ratio (around 1.4) along the entire genome, as was previously shown^15^, in the aberrant samples the expected change in the allelic ratio in the duplicated chromosome was easily observed (Fig. 1b and Supplementary Fig. 2). Statistical significance was calculated with a one tailed *t*-test comparing the SNPs major/minor ratio values in each window with the total SNP pool and false discovery rate (FDR) correcting for multiple testing. Importantly, the observed change in the allelic ratio was highly statistically significant (Fig. 1b and Supplementary Fig. 2). This method was sensitive enough to detect the duplication of chromosome 1q in a sample with relatively low sequencing depth (∼6 × 10^6^ mapped reads).

**Figure 3:**
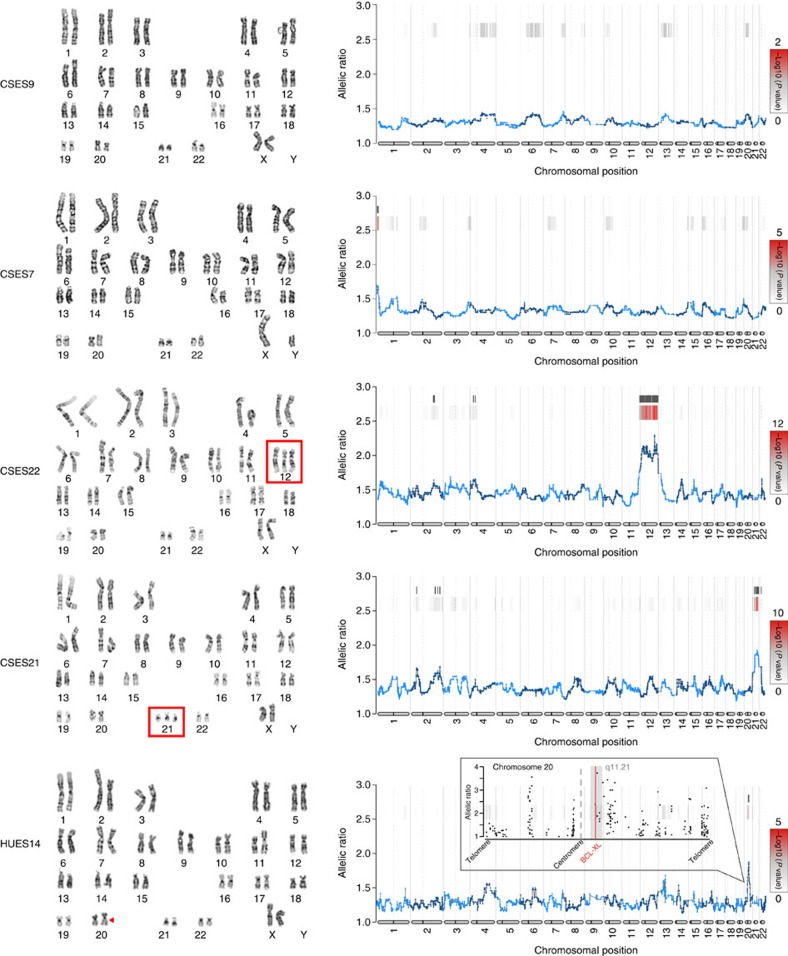
eSNP-Karyotype and G-banding karyotype analyses of hESC samples. G-banding staining of different hESCs cell lines alongside eSNP-Karyotyping analysis of the same cell lines. Widow size for the moving median plots is 151 SNPs except for the HUES14 cell line were window of 51 SNPs was used. In addition, for HUES14 cell line, only common SNPs were used for the analysis. The inset in the HUES14 eSNP-Karyotyping shows enlargement of chromosome 20, and the ratio between the major to minor allele of each expressed common SNP. hESC, human ESC.

To further validate our method, we extracted RNA from five different cell lines (CSES9, CSES7, CSES22, CSES21 and HUES14), all samples were analysed by RNA-seq, followed by eSNP-Karyotyping and by the gold standard G-banding karyotype. As shown in Fig. 3, two of the samples did not show any detectable chromosomal aberration, while in other two samples we could identify chromosomal aberrations in either chromosome 12 or 21, by both G-banding karyotype and eSNP-Karyotyping. In HUES14 cell line, we could detect significant signal in a small region of chromosome 20. This potential CNV contains a region (q11.21), which is well known for providing selective advantage to hPSC, due to the duplication of the anti-apoptotic gene *BCL-XL*^18^,^19^,^20^,^21^. Importantly, difference in size between the two copies of chromosome 20 was also visible by G-banding. This analysis supports the validity of the eSNP-Karyotyping methodology.

Since eSNP-Karyotyping, as opposed to e-Karyotyping, does not require a corresponding diploid sample as a baseline, it performs better with samples from differentiated cells where differences in the extent of differentiation may cause differences in gene expression between samples. For example, there are only two studies with RNA-seq samples of differentiated pancreatic progenitor cells^22^,^23^. Analysis of these data sets by e-Karyotyping is extremely noisy due to differences in gene expression patterns between the studies (Fig. 1c and Supplementary Fig. 3a). However, using eSNP-Karyotyping, we could easily detect trisomy 12 and 17 in embryonic stem cell (ESC)-derived samples from one of the studies (Fig. 1d and Supplementary Fig. 3b).

eSNP-Karyotyping can also perform successfully on mouse samples as long as their origin is outbred mice. Reports on stimulus-triggered acquisition of pluripotency^24^ were re-evaluated by multiple analyses, including analysis of the genomic integrity of the samples using comparisons between the published CHIP-Seq data^25^. Here we used the gene expression data to analyse the chromosomal integrity by eSNP-Karyotyping. We thus could validate the existence of trisomies 6 and 11 in the trophoblast stem cell samples (Supplementary Fig. 4a). Adding to the original analysis, we could also show that the epiblast stem cell samples, which did not have a CHIP-Seq profile, had trisomy 13 and a probable mosaic trisomy 8 (Supplementary Fig. 4b). The stimulus-triggered acquisition of pluripotency cells were diploid as reported (Supplementary Fig. 4c).

Detection of chromosomal aberrations in small chromosomes can be more challenging. Analysis of expression data from fibroblasts of Down's syndrome patient could successfully detect trisomy 21 (Supplementary Fig. 5). However, on reprogramming of these sample into induced pluripotent stem cells, we could detect an additional trisomy in chromosome 20 (Supplementary Fig. 5). Importantly, e-Karyotyping did not detect this aberration in a clear manner^26^.

**Figure 4:**
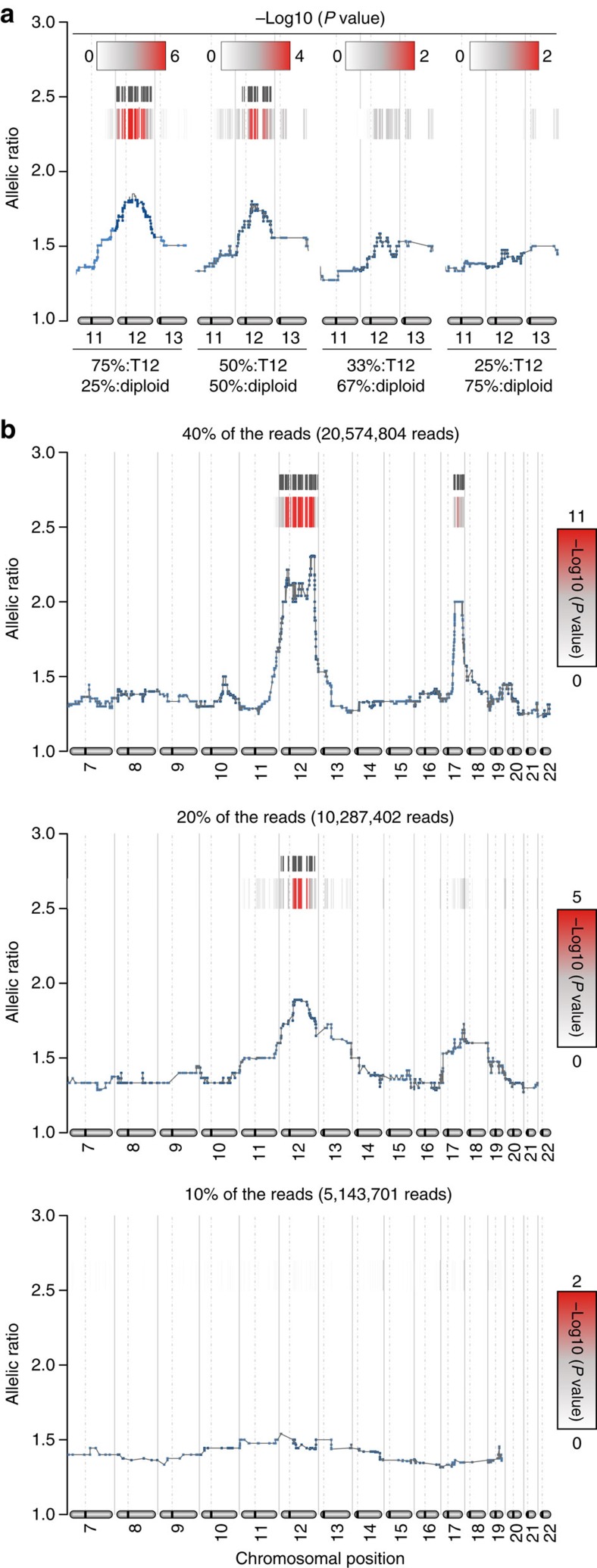
The effect of read number and population composition on eSNP-Karyotyping sensitivity. (**a**) Sensitivity analysis of the eSNP-Karyotyping method. Reads from the sample described in the upper panel of Fig. 1b were mixed, in different ratios, with diploid sample from the same study, and analysed with eSNP-Karyotyping. Only the relevant genomic regions are shown. (**b**) Assessment of the number of reads needed for significant detection of chromosomal duplications. Different numbers of reads from the sample shown in Fig. 1d were randomly selected and tested with eSNP-Karyotyping.

eSNP-Karyotyping detection power depends on the population diversity and the reading depth. In a mixed population of diploid and aneuploid cells, the detection power is noticeably reduced. To assess the necessary percentage of aneuploid cells in a population for a reliable detection of a trisomy (that is, the degree of mosaicism that could be detected), we mixed reads from two neural samples with either diploid or trisomy 12, both from the same study^27^. When half of the sequencing reads originated from the aberrant samples, the trisomy was still easily detected. However, when only a third of the reads were from trisomy 12 samples, the trisomy was visible, though not statistically significant (Fig. 4a). To determine the necessary read number, we used the pancreatic progenitor sample, which has a high reading number, and gradually reduced the number of reads^23^. This assessment showed that 15–20 × 10^6^ mapped reads allow for good detection power of chromosomal aberrations, with ∼2,000 detected SNPs (Fig. 4b and Supplementary Fig. 6).

### Analysing loss of heterozygosity using eSNP-Karyotyping

**Figure 5:**
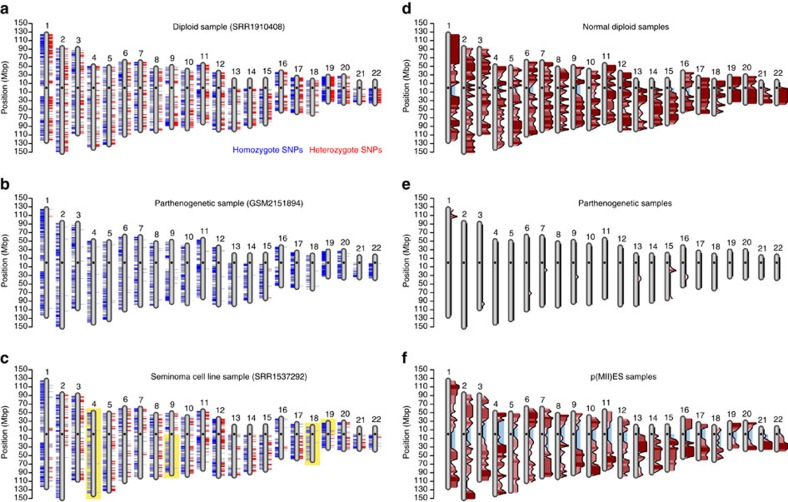
LOH detection and recombination mapping using RNA-Seq data. (**a**–**c**) LOH analysis in normal ESCs (**a**), parthenogenetic ESCs (**b**) and a seminoma cell line (**c**). Blue lines represent expressed homozygous SNPs and red lines represent expressed heterozygous SNPs. Colour intensity represents the SNP density within a specific region. Regions of statistically significant LOH are highlighted with a yellow background (**a**,**c**). (**d**–**f**) Heterozygosity map constructed from four samples of normal ESCs (**a**), parthenogenetic ESCs (**b**) and p(MII)ES cells (**c**) samples. The red bars show the likelihood of each 5 Mb to be heterozygous. Light blue background highlights homozygosity regions around the centromeres.

To identify loss of heterozygosity (LOH, deletions or uniparental disomies), we took a complementary approach. We reasoned that in these cases, all genes should show monoallelic expression since they only exist in one copy or two duplicated copies. For this analysis, we obtained a list of the common SNP positions in the human genome from the dbSNP database^28^. First, we filtered all common SNP positions below the sequencing coverage of 20 reads. Then, we intersected the list of SNPs detected in the duplication analysis with the dbSNP list. In this manner, we determined whether each expressed known SNP position was heterozygote or homozygote. Finally, we examined the distribution of the homozygous and heterozygous SNPs along the genome (Fig. 2). For each chromosomal arm, the ratio of homozygote to heterozygote SNPs was calculated and compared with the ratios of the rest of the arms using *t*-test. Homozygous arms are those with FDR-corrected *P* value bellow 0.001 and homozygote to heterozygote ratio five times greater than this proportion for all the autosomal chromosomes. The diploid samples showed an equal distribution of homozygous and heterozygous SNPs along the genome (Fig. 5a). However, parthenogenetic ESCs (pESCs), which originated from an activated oocyte and have a duplicated maternal genome, showed a complete monoallelic expression, confirming the validity of the method (Fig. 5b). The seminoma TCam-2 cell line sample, which is a germ cell tumour, showed regions of homozygosity in variable sizes up to an entire chromosome (Fig. 5c), suggesting LOH events in this sample^29^.

To determine the necessary number of reads required for clear observation of LOH, we sampled different numbers of reads-out of the original data set (which contains ∼55 × 10^6^ mapped reads) and performed our analysis on the read-depleted files. The observed aberration was still easily detected even with 50% of the reads (Supplementary Fig. 7). However, reducing the number of reads to 25% abolished the effectiveness of the technique, since the number of heterozygous known SNPs, with coverage above 20 reads, was not sufficient for a definitive conclusion (Supplementary Fig. 7). This analysis indicated that ∼20 × 10^6^ can give good detection power of LOH.

### Mapping meiotic recombination using eSNP-Karyotyping

Finally, we decided to map meiotic recombination from RNA-Seq data with this methodology. During oocyte development, homologous chromosomes exchange segments by homologous recombination. Then, homologous chromosomes are separated during meiosis-I followed by separation of sister chromatids during meiosis-II. Examination of zygosity patterns of oocytes or pESCs that failed in chromosome segregation during meiosis-I or meiosis-II (p(MII)ES) can reveal sites where homologous recombination occurred^30^. In humans, only one study has been published, which analysed only one sample of p(MII)ES^31^. Here, we analysed four samples of pESCs, four diploid ESCs and four p(MII)ES cells, with approximately the same number of reads^32^,^33^ (Supplementary Fig. 6). The p(MII)ES (also called SWAP cells) originates from activation of oocytes that failed to extrude the polar body^34^. We performed an analysis that searched for heterozygosity in blocks of 5 Mb along the genome. Blocks with fewer than three heterozygous SNPs were defined as homozygous, whereas blocks with three or more were defined as heterozygous. We thus mapped the zygosity state of each sample (Supplementary Fig. 8). Then we plotted a histogram along each chromosome that determines the likelihood of each block to be heterozygous in each group of samples. The pESCs showed almost no regions of heterozygosity whereas the normal ESCs showed heterozygosity along the entire chromosome length (Fig. 5d,e). Interestingly, near the centromeres of the p(MII)ES cells, we observed relative homozygosity, and the likelihood for heterozygosity increased as the region got closer to the telomeres, indicating a lack of recombination in this region (Fig. 5f).

## Discussion

Chromosomal aberration analysis using gene expression data can prove valuable for assuring a normal karyotype or to detect major chromosomal aberrations. Unlike traditional DNA-based methods, this method is mainly designated for studies where gene expression analysis by RNA-Seq was performed for other purposes, and the expression data are already available and can be utilized for genomic integrity analysis as well.

The observed allelic ratio in the diploid chromosomes, which was constantly around 1.4, is similar and even slightly lower than the ratio found in a recent report^15^. A few factors contribute to this ratio: (1) monoallelic or biased expression of certain genes, due to different genetic and epigenetic status that can affect their expression; (2) genes expressed at low levels may show some allelic bias when analysed by RNA-Seq as a result of the low number of reads; and (3) higher chances of a read that contains the reference SNP to be mapped to the reference genome then for read that contains the alternative SNP. This well-known phenomenon may be partially overcome by different methodologies^35^,^36^. Still, in a recent report that analysed the transcriptome of embryonic stem cells the ratio was over 1.7 even with the use of a methodology to overcome the reference genome bias^15^.

Each of the current genomic integrity analysis techniques has its strengths and limitations regarding genomic aberration detection^10^. In terms of sensitivity, cytogenetic methods are the most sensitive as they are performed on single metaphase spreads. However, SNP arrays, CGH arrays, e-Karyotyping and eSNP-Karyotyping are comparable in terms of sensitivity since they are performed on cell populations. When analysing cells in culture, if the aberration provides a selective advantage to the cells it will rapidly take over the culture; however, if they are neutral or harmful they are much less likely to fixate in the population^5^. In terms of resolution, WGS has the highest performance followed by the array-based methods^10^. e-Karyotyping was shown to have a resolution similar to SKY and GIEMSA banding^6^,^10^; however, it can vary as a function of multiple parameters such as the diploid baseline for comparison and the platform used for gene expression analysis^10^. eSNP-Karyotyping resolution is heavily dependent on the sequencing depth and genome composition. For this reason we limited our analysis to the entire chromosome or chromosome arm. The cost and duration of WGS is much higher than SNP and CGH arrays, which are comparable to cytogenetic-based methods^10^. However, gene expression-based techniques are performed on data obtained for other purposes such as differential gene expression analysis, so the cost is not devoted entirely to genomic integrity assay. Similar to SNP arrays, CGH arrays and e-Karyotyping, eSNP-Karyotyping cannot identify balanced translocations.

Although the expression-level-based method, e-Karyotyping, is already successfully used^2^,^6^,^7^,^8^,^9^, eSNP-Karyotyping may have a few advantages: (1) as opposed to e-Karyotyping, eSNP-Karyotyping does not require any additional normal samples other than the sample for examination, which makes the analysis quicker and easier. In cases where the gene expression profile of the diploid matched sample is not available, genomic integrity analysis using e-Karyotyping cannot be performed. (2) eSNP-Karyotyping works well with small chromosomes, as shown with the trisomy 21 in the Down's syndrome patient. (3) Since there is no need for comparison to normal samples, it can be used to study chromosomal aberrations in samples with multiple different aberrations such as cancer cells, as long as the population is homogenous. (4) Since eSNP-Karyotyping is based on the allelic ratio and not on expression levels, aberrations that cause profound changes in gene expression in the entire genome will be detected by eSNP-Karyotyping.

Analysis of allelic expression from expressed alleles can be utilized for studying epigenetic phenomena. Some of the potential uses include studying monoallelic expression, following the process of X inactivation in female cells by analysing heterozygosity along the X chromosome or detecting aberrations in imprinted genes. We believe that eSNP-Karyotyping can prove helpful in the analysis of the genetic integrity of pluripotent stem cells and their derivatives in addition to other fields of genetic research.

## Methods

### e-Karyotyping analysis

The data were analysed as previously described for microarray data sets^2^,^6^,^7^,^10^. Illumina Gene expression RNA-Sequencing profiles were obtained from the SRA (http://www.ncbi.nlm.nih.gov/Traces/sra/) database^11^. The SRA files were extracted using SRAtools^11^ and aligned to HG38 reference genome using TopHat2 (ref. 12) allowing only one alignment per read. Cufflinks^13^ was used to obtain normalized FPKM values for each sample. The following analysis was performed in batches according to the cell type or study. In each analysis, the samples were merged into a single table and the transcripts were organized by their chromosomal location. Expression values of zero were set to 10^−7^ to allow log 2 transformation of all the expression values. Next, samples with an expression value below 1 FPKM were adjusted to 1 to enable statistical testing. We considered transcripts with an expression level of 1 FPKM as unexpressed. Transcripts unexpressed in more than 20% of the samples were removed to decrease expression noise. In each analysis batch, the median expression of a transcript across the entire batch was subtracted from the expression value of each transcript in each sample, to obtain a comparative value. This median then served as the baseline for examining expression bias. To reduce noise, the sum of squares of the relative expression values was calculated for each transcript and the 10% most variable genes were removed from further analysis. The data were processed and visualized using a CGH analysis software programme, CGH-Explorer^14^ (http://heim.ifi.uio.no/bioinf/Projects/CGHExplorer/). Gene expression regional bias was detected using the piecewise constant fit algorithm, using a set of parameters as follows: least allowed deviation=0.25; least allowed aberration size=50; Winsorize at quantile=0.001; penalty=12; and threshold=0.01. Moving-average plots were drawn using the moving-average fit tool, with windows of 200 genes.

### Detection of chromosomal duplications using eSNP-Karyotyping

BAM files were edited using Picard tools and SNPs were called using the GATK HaplotypeCaller. The SNPs were filtered according to the reading depth and allelic frequency to reduce errors and noise. SNPs with low coverage (below 20 reads) or with low minor allele frequency in the total allele poll (lower than 0.2) were discarded. Next, for each SNP, the major to minor frequency ratio was calculated and the table was sorted by the chromosomal position. For visualization, moving medians of the major to minor ratios were plotted along the moving medians of the chromosomal positions. Usually, a window of 100–150 SNPs was used. The *P* value was calculated with a one tailed *t*-test comparing the SNPs major/minor values in the window to the total SNP pool and correcting for multiple testing using FDR correction. In specific cases, to reduce noise, the list of SNPs was further filtered to contain only known SNPs. For the sensitivity assay, reads from diploid (SRR1561108) and trisomy 12 (SRR1561105) samples, from the same study, were mixed in different ratios using the SAMtools view and merge functions. To determine the necessary read number, different percentages of reads, from 10 up to 100% were randomly selected and analysed using eSNP-Karyotyping. The sample selected for this assay had trisomies 12 and 17 (SRR1693240), and covered with more than 50M mapped reads. The entire workflow and visualization of the data were performed using R statistical software (http://www.r-project.org/).

### Detection of LOH using eSNP-Karyotyping

A list of common SNPs in the human genome was obtained from the dbSNP database (http://www.ncbi.nlm.nih.gov/SNP/). For each common SNP we first determined whether it was homozygote or heterozygote by checking whether it was detected as a valid SNP in our SNP calling. Next, SNPs that were covered by fewer than 20 reads were discarded. The reading depth for each SNP was determined by the SAMtools depth function. For each chromosome we calculated the number of homozygote and heterozygote SNPs in blocks of 1.5 Mb and plotted them along the chromosome. The entire workflow and visualization of the data were performed using R. To obtain *P* value, we determined the ratio of the number of homozygote to heterozygote SNPs for each chromosome arm. Then, we determined for each arm if this ratio is statistically different from the rest of the chromosome arms by *t*-test. The *P* value list was corrected for multiple testing using FDR correction. True LOH is considered as an arm with *P* value lower than 0.001 and a homozygote to heterozygote SNPs ratio five times greater than the ratio of all the autosomal chromosomes.

### Mapping recombination using eSNP-Karyotyping

Four normal ESC samples, four parthenogenetic ESC samples and four p(MII)ESC samples were used in the analysis. All 12 samples had ∼20 × 10^6^ mapped reads. For each sample we calculated the number of homozygote and the number of heterozygote SNPs in blocks of 5 Mb. A block was considered informative if it contained at least three homozygote SNPs. Informative blocks were considered homozygote if they contained fewer than three heterozygote SNPs. These parameters were selected because they allow for low positive calls in the parthenogenetic cells, identification of putative homozygous regions in the SWAP samples, and a high percentage of informative overlapping blocks between the samples. Next, for each group of four samples, we plotted their likelihood of being heterozygote in each block. The entire workflow and visualization of the data were performed using R.

### Cell culture

Human ESCs (CSES^37^,^38^ and HUES14 (ref. 39)) were cultured on mouse embryonic fibroblast treatment with mitomycin-C. Culture medium contained KnockOut Dulbecco's modified Eagle's medium (Gibco-Invitrogen, CA) supplemented with 15% KnockOut-SR (Gibco-Invitrogen, CA), 1 mM glutamine, 0.1 mM β-mercaptoethanol (Sigma-Aldrich, MO), 1% non-essential amino-acid stock (Gibco-Invitrogen, CA), penicillin (50 U ml^−1^), streptomycin (50 μg ml^−1^), and 8 ng ml^−1^ fibroblast growth factor 2 (Gibco-Invitrogen, CA). Cells were passaged using trypsin-EDTA (Biological Industries, Beit Haemek, Israel).

### RNA extraction and sequencing

Total RNA was extracted using NucleoSpin RNA Plus kit (Marcherey–Nagel). RNA integrity (RIN>9) was validated using Bioanalyzer (Agilent Technologies). mRNA was enriched by Poly-A selection, and sequencing libraries were prepared using TruSeq RNA Library Prep Kit v2 (Illumina). Single-end 85 bp sequencing was performed using Illumina Next-Seq500.

### G-banding

Before cell harvesting, Colcemid (Invitrogen) was added directly to the plate of cells, at a final concentration of 100 ng ml^−1^ for 40 min. Then, cells were trypsinized, treated with hypotonic solution for 20 min and fixed. Metaphases were spread on microscope slides, and using standard G-banding staining chromosomes were classified according to the International System for Human Cytogenetic Nomenclature.

### Code availability

eSNP-Karyotyping R package is available for download from GitHub (https://github.com/BenvenLab/eSNPKaryotyping)

### Data availability

Sequencing data performed for this study were deposit in Gene Expression Omnibus (GEO) under the accession number GSE81402.

## Additional information

**Accession codes:** Sequencing data performed for this study were deposit in Gene Expression Omnibus (GEO) under the accession number GSE81402.

**How to cite this article:** Weissbein, U. *et al*. Analysis of chromosomal aberrations and recombination by allelic bias in RNA-Seq. *Nat. Commun.* 7:12144 doi: 10.1038/ncomms12144 (2016).

## Supplementary Material

Supplementary Information Supplementary Figures 1-8 and Supplementary Table 1
