# Supplementary material for: Analysis of chromosomal aberrations and recombination by allelic bias in RNA-Seq
Source: Nat Commun. 2016 Jul 7;7:12144. doi: 10.1038/ncomms12144 (PMC4941052; doi:10.1038/ncomms12144)
Supplement: Supplementary Information — Supplementary Figures 1-8 and Supplementary Table 1 [file ncomms12144-s1.pdf]

Supplementary Figure 1

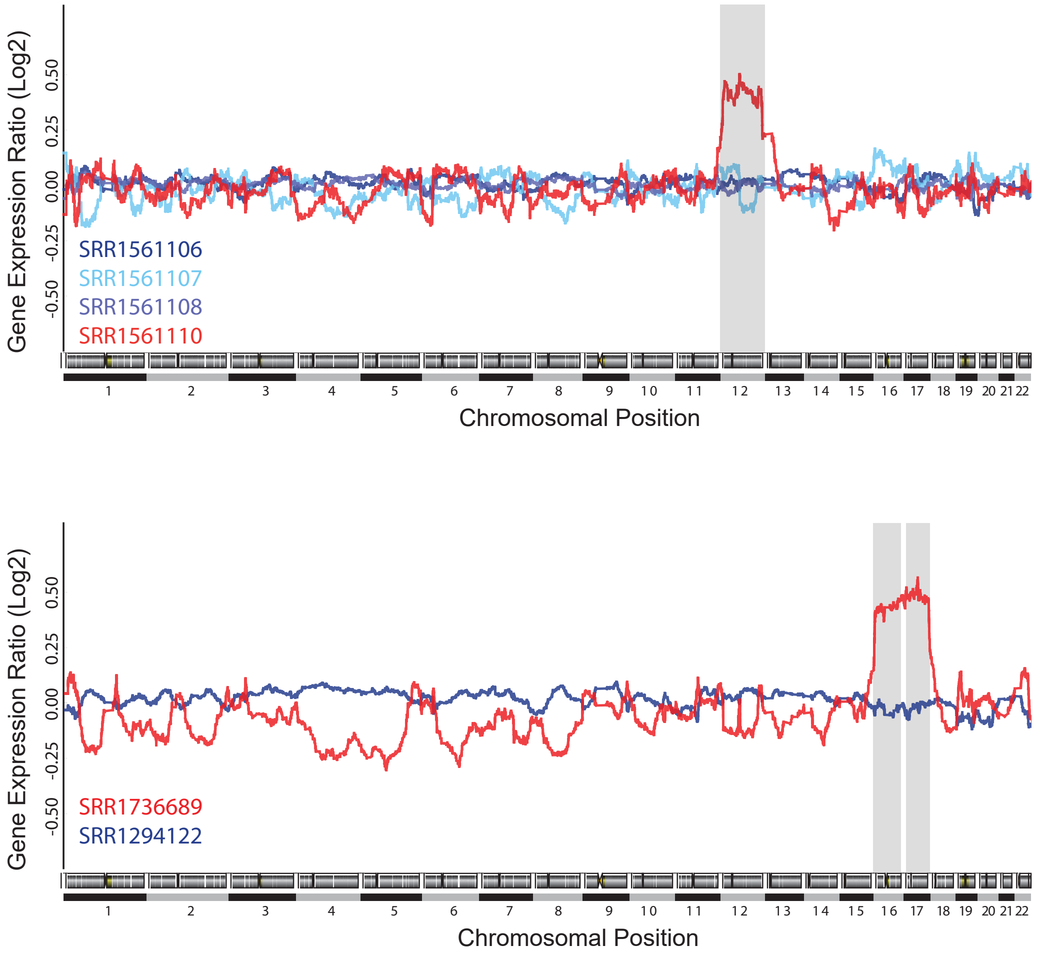

Detection of chromosomal duplications by e-Karyotyping using RNA-Seq data. Shown are moving average plots of the second repeats of the examples from Figure 1a. The gray background represents aneuploidy as identified by the PCF algorithm.

Supplementary Figure 2

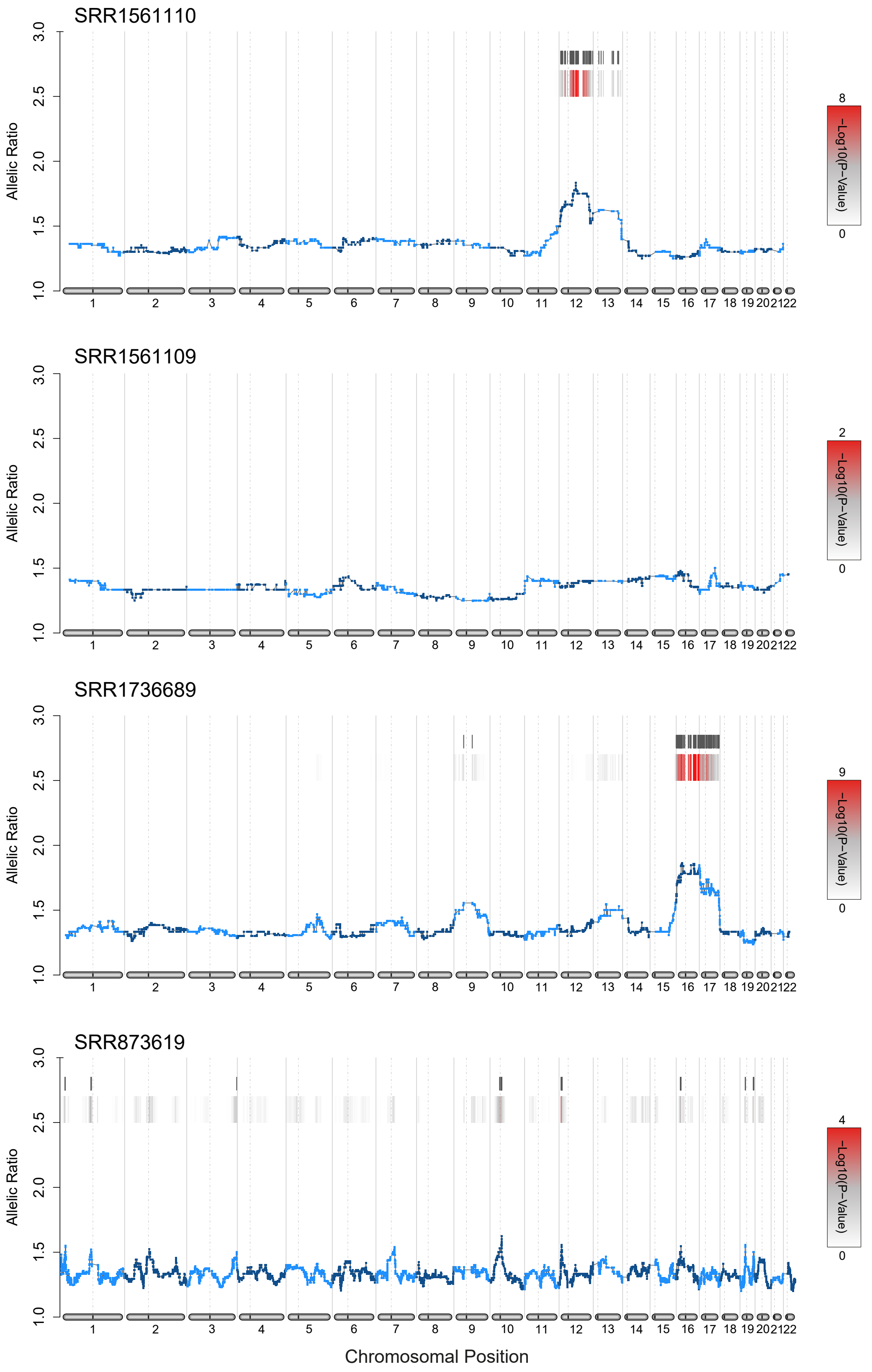

Detection of chromosomal duplications by eSNP-Karyotyping using RNA-Seq data. Shown are moving average plots of the second repeats of the examples from Figure 1a and the diploid samples. Color bars represent FDR corrected p-values. Positions with a p-value lower than 0.01 are marked by black lines.

Supplementary Figure 3

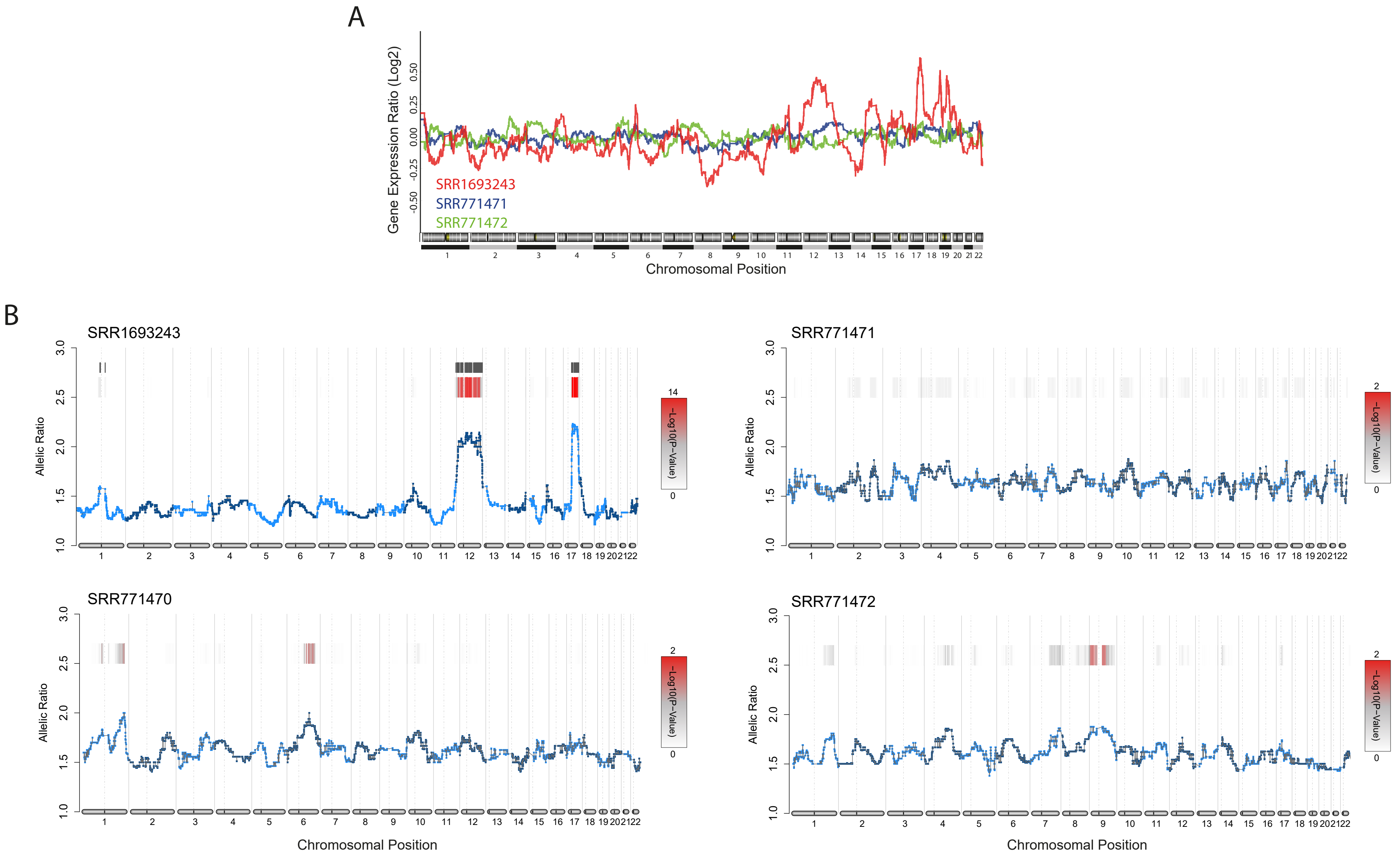

Karyotypic analysis of PSC-derived pancreatic progenitor cells. (a) e-Karyotyping of additional pancreatic progenitor samples. Shown are moving average plots. **(b)** eSNP-Karyotyping of additional pancreatic progenitor samples.

Supplementary Figure 4

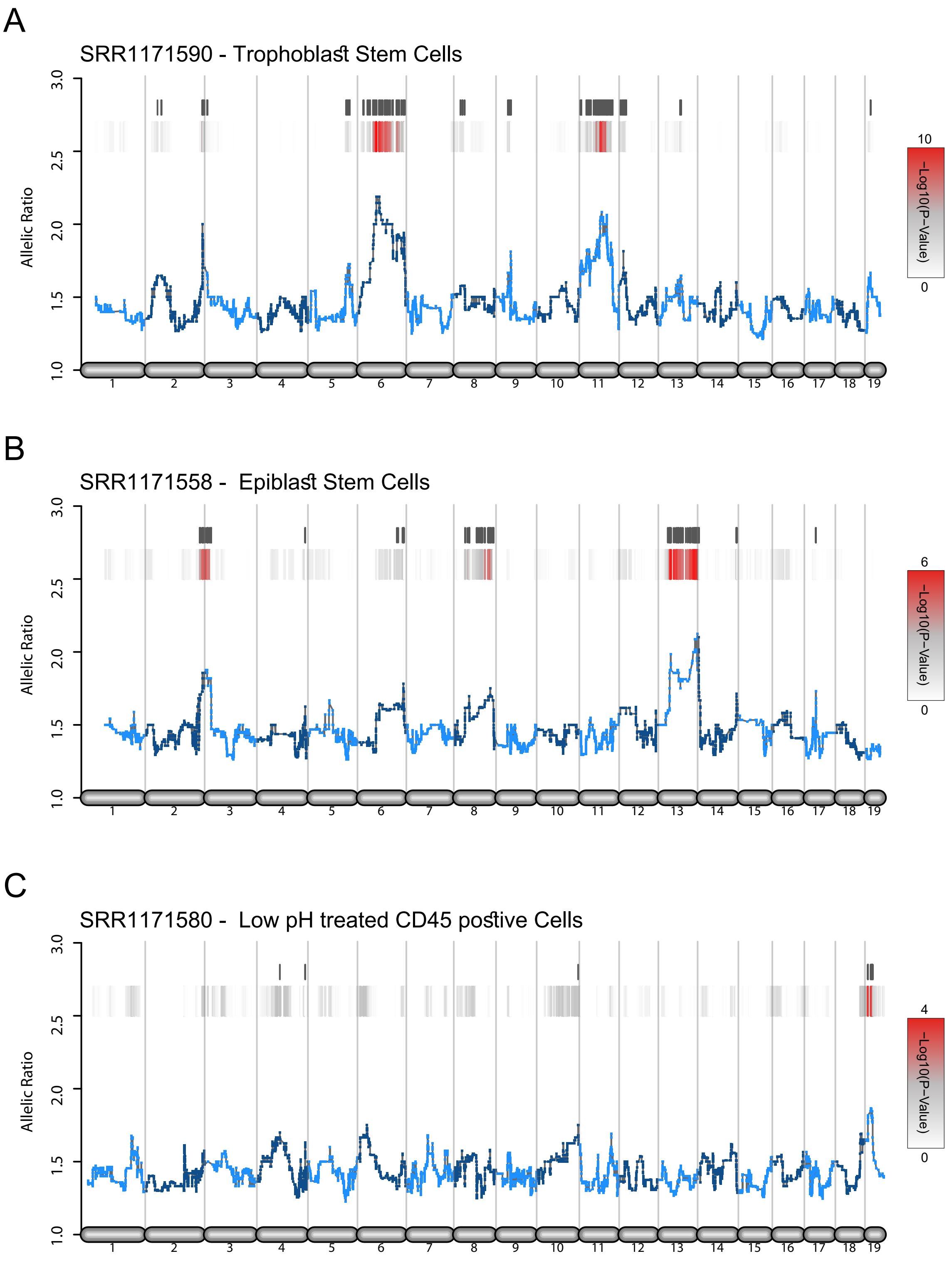

eSNP-Karyotyping Analysis of samples from the STAP study. Shown are the two aberrant samples (**a**,**b**) and an additional diploid sample (**c**).

Supplementary Figure 5

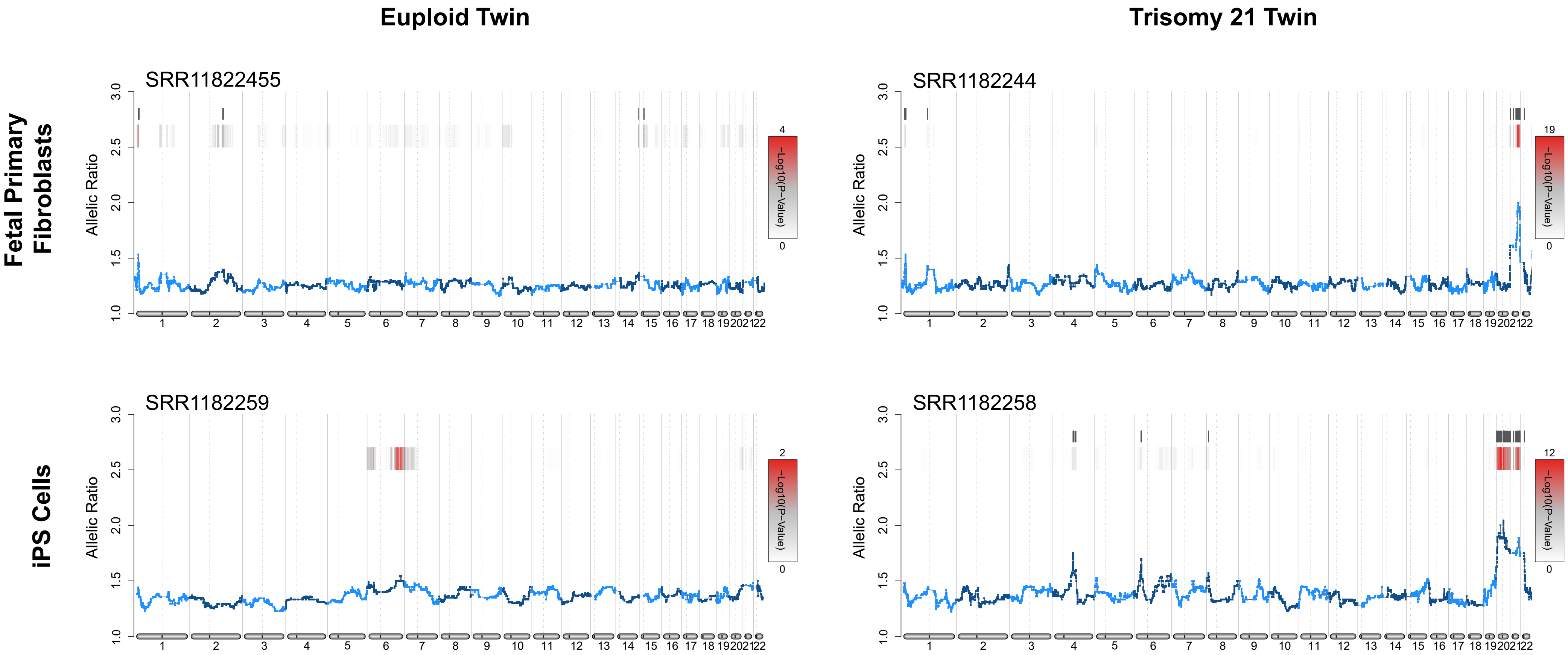

Analysis of fibroblasts and iPSCs from diploid and Down syndrome twins. Shown are eSNP-Karyotyping analysis with common SNPs alone.

Supplementary Figure 6

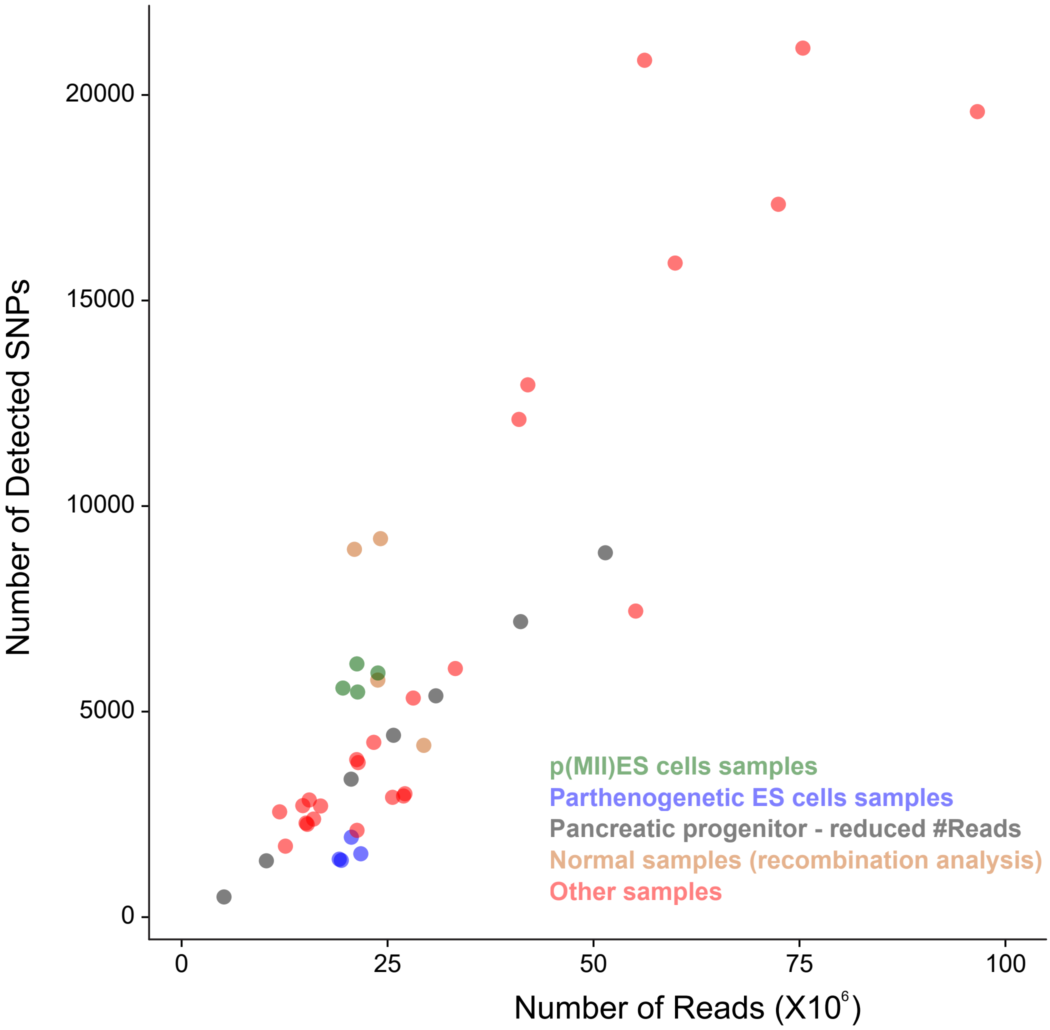

The effect of the sequencing depth on the number of detected SNPs. For each analyzed sample, the number of mapped reads and the number of detected SNPs are plotted. For this analysis, equal algorithm parameters were applied for all the samples (minimum coverage per SNP=20 and minimal frequency of the minor allele=0.2).

Supplementary Figure 7

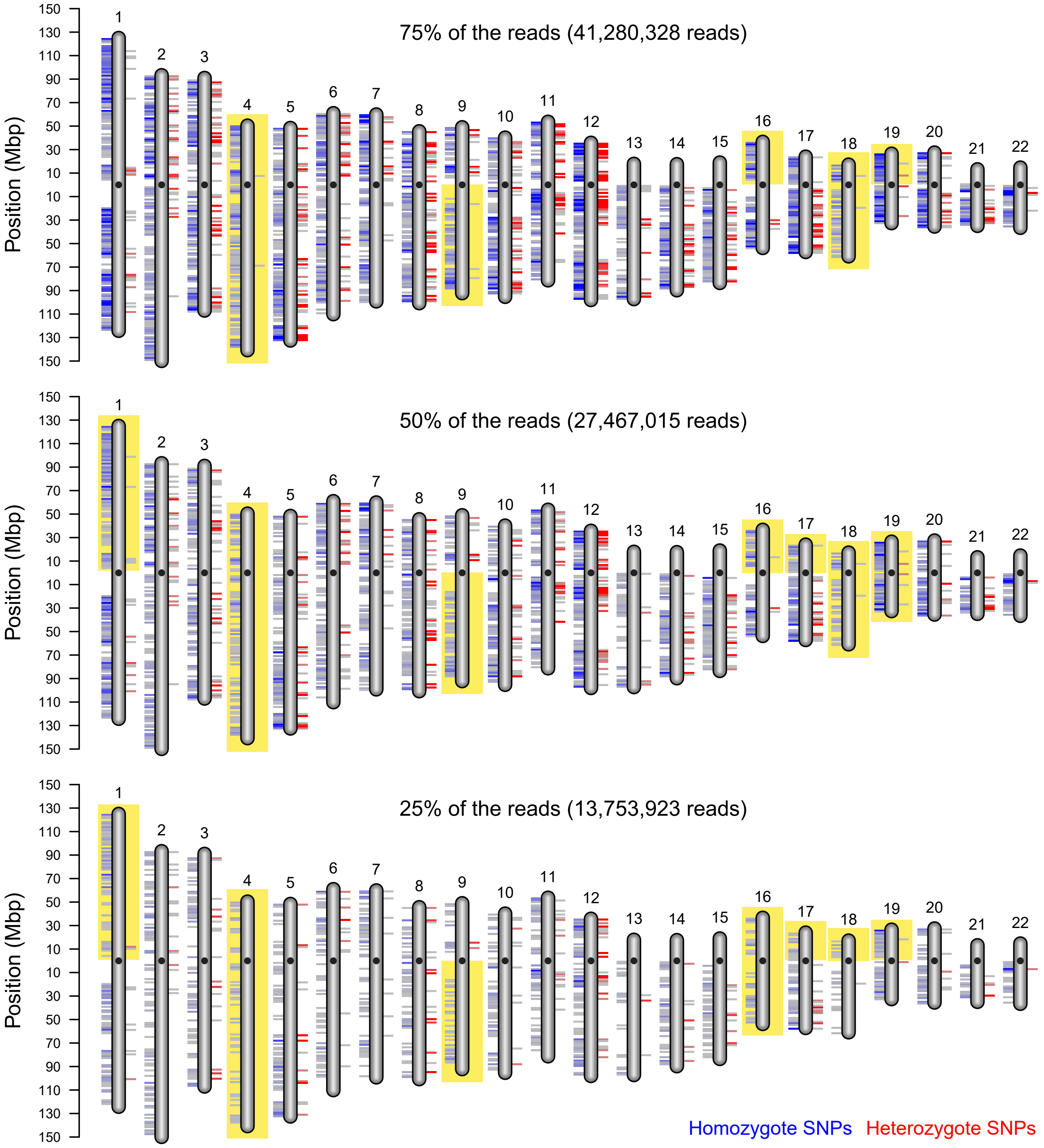

Reads number effect on LOH detection. Assessment of the number of reads necessary for detection of LOH. Different numbers of reads from the sample shown in Fig. 3c (SRR1537292) were randomly selected and tested with the eSNP-Karyotyping LOH detection procedure. Blue lines represent expressed homozygous SNPs and red lines represent expressed heterozygous SNPs. Color intensity represents the SNP density within a specific region. Regions of LOH detected with 100% of the reads are highlighted by the yellow background.

Supplementary Figure 8

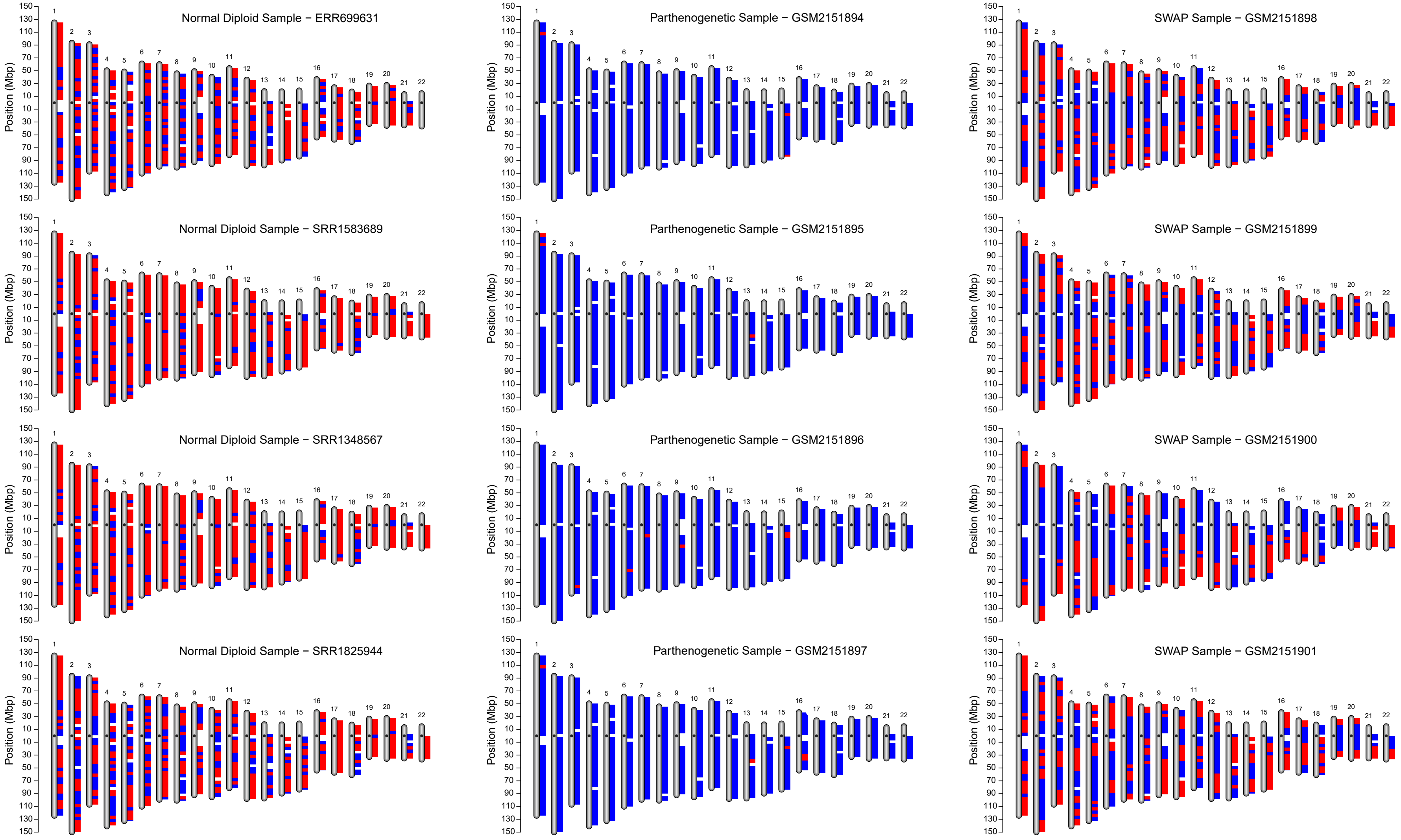

Zygosity maps of multiple different samples. Shown is an analysis of 4 parthenogenetic ESC samples, 4 p(MII)ES samples and 4 normal ESC samples. The number of known SNPs in blocks of 5MB was counted. Block with fewer than 3 heterozygous SNPs were considered homozygote (blue), whereas blocks with 3 or more heterozygous SNPs were considered heterozygous (red). Blocks with fewer than 3 expressed homozygous SNPs were considered unexpressed blocks (white)

Supplementary Table 1: Samples used in the study:

Sample	Karyotype	Description	# Mapped Reads	Layout
SRR1561105	T12	Neuronal progeny differentiated from FTD3#6-iPSC	16,611,124	Single
SRR1561106	Diploid	Neuronal progeny differentiated from H9-hESC	18,474,516	Single
SRR1561107	Diploid	Neuronal progeny differentiated from H9-hESC	16,019,672	Single
SRR1561108	Diploid	Neuronal progeny differentiated from FTD3#6-PGRN	17,267,890	Single
SRR1561109	Diploid	Neuronal progeny differentiated from FTD3#6-PGRN	15,105,887	Single
SRR1561110	T12	Neuronal progeny differentiated from FTD3#6-iPSC	15,248,161	Single
SRR1736689	T16T17	Conventional WIS2 hESC, rep1	13,956,488	Single
SRR1736690	T16T17	Conventional WIS2 hESC, rep2	14,061,546	Single
SRR873619	Diploid	Human WT-33 iPSC-1 A	56,197,759	Paired
SRR1294122	Diploid	Human ES cell line UCLA6 (ucla6)	34,105,083	Single
SRR061728	T1q	Human ES h1 cells, preimmune	6,217,527	Single
SRR1015684	Diploid	PGP9-iPSCs	26,267,171	Single
SRR1693240	T12T17	Sorted Pdx1-EGFP+ pancreatic progenitors	49,215,988	Single
SRR1693243	T12T17	Sorted Pdx1-EGFP+ pancreatic progenitors	51,959,891	Single
SRR771470	Diploid	Pancreatic progenitors cells	24,501,954	Paired
SRR771471	Diploid	Pancreatic progenitors cells	35,899,518	Paired
SRR771472	Diploid	Pancreatic progenitors cells	21,097,662	Paired
SRR1182244	T21	Twin 1_T21_fetal primary fibroblasts	286,485,960	Paired
SRR1182258	T20T21	Twin 1_T21_iPSCs	96,595,744	Paired
SRR1182245	Diploid	Twin 2_euploid_fetal primary fibroblasts	275,195,598	Paired
SRR1182259	Diploid	Twin 2_euploid_iPSCs	62,479,368	Paired
SRR1910408	Diploid	hESC neural differentiation at days 8	37,087,726	Single
SRR1537292	d2q,d4,d9q,d18	Seminoma cell line	55,119,965	Single
ERR699631	Diploid	Definitive endoderm differentiated from H9 stem cells	23,810,950	Single
SRR1583689	Diploid	Nuclear-transfer embryonic stem cells	20,976,618	Single
SRR1348567	Diploid	IMR90 iPS	24,141,429	Single
SRR1825944	Diploid	H1 human embryonic stem cells derived ectoderm	29,393,979	Single
GSM2151894	Parthenogenetic	swaPS2 heterologous genome exchange parthenogenetic stem cell line	20,599,574	Single
GSM2151895	Parthenogenetic	pES2 Human parthenogenetic ES cells	19,130,452	Single
GSM2151896	Parthenogenetic	pES7 Human parthenogenetic ES cells	21,751,515	Single
GSM2151897	Parthenogenetic	pES10 Human parthenogenetic ES cells	19,381,744	Single
GSM2151898	Diploid	swaPS1 heterologous genome exchange parthenogenetic stem cell line	19,579,643	Single
GSM2151899	Diploid	swaPS6 heterologous genome exchange parthenogenetic stem cell line	23,831,139	Single
GSM2151900	Diploid	swaPS9 heterologous genome exchange parthenogenetic stem cell line	21,364,504	Single
GSM2151901	Diploid	swaPS10 heterologous genome exchange parthenogenetic stem cell line	21,273,489	Single
GSM2151889	Diploid	CSES9 Human embryonic stem cells	27,402,128	Single
GSM2151890	Diploid	CSES7 Human embryonic stem cells	17,626,561	Single
GSM2151891	T12	CSES22 Human embryonic stem cells	38,394,206	Single
GSM2151892	T21	CSES21 Human embryonic stem cells	44,996,020	Single
GSM2151893	d20q21.11	HUES14 embryonic stem cells	36,906,683	Single
